# Optimizing alpha-amylase from *Bacillus amyloliquefaciens* on bread waste for effective industrial wastewater treatment and textile desizing through response surface methodology

**DOI:** 10.1038/s41598-023-46384-6

**Published:** 2023-11-06

**Authors:** Basma T. Abd-Elhalim, Rawia F. Gamal, Salwa M. El-Sayed, Samah H. Abu-Hussien

**Affiliations:** 1 Department of Agricultural Microbiology, Faculty of Agriculture, Ain Shams University, Hadayek Shoubra, P.O. Box 68, Cairo, 11241 Egypt; 2 Department of Biochemistry, Faculty of Agriculture, Ain Shams University, Hadayek Shoubra, P.O. Box 68, Cairo, 11241 Egypt

**Keywords:** Biochemistry, Biochemistry, Microbiology

## Abstract

Food waste is a major issue, with one-third of food wasted yearly. This study aimed to produce sustainably the industrial enzyme alpha-amylase using discarded bread waste. Brown (BBW) and white bread waste (WBW) were tested as growth substrates using solid-state and submerged fermentation. The biosynthesized α- amylase is applied to treat starch-heavy industrial wastewater and for textile desizing. *Bacillus amyloliquefaciens* showed the highest starch hydrolysis and enzyme activity on solid and liquid media. α-amylase production by *B. amyloliquefaciens* was optimized via a one-factor-at-a-time evaluation of production parameters. Optimal production occurred by submerged fermentation of BBW inoculated with 2% *B. amyloliquefaciens* at 37 °C and 200rpm for 24 h, reaching 695.2 U/mL α- amylase. The crude enzyme was immobilized on calcium alginate beads with 96.6% efficiency and kept 88.5% activity after 20 reuses, enhancing stability. A Box–Behnken design (BOX) assessed variable interactions. Response surface methodology (RSM) generated a quadratic model and analysis of variance (ANOVA analysis) fitting experimental starch hydrolysis data. Optimal conditions were pH 9, 45 °C, 70% starch, and 27.5 U/mL enzyme incubated for 15 min of contact time, with a high R^2^ of 0.83. ANOVA confirmed the enzyme's alkaliphilic and thermophilic nature. Using enzyme concentrations ranging from 10.9 to 695.1 U/mL, the enzyme desized textiles in 15 min at pH 9.0 and 45 °C with 96.3% efficiency. Overall, the optimized α- amylase from bread waste has industrial potential for sustainable starch processing.

## Introduction

Food waste is a major global issue, with nearly one-third of food produced going to waste each year. Bread products, in particular, contribute substantially to food waste streams. Food waste is characterized as food loss by consumers or merchants because of inadequate storage, a lack of awareness, and bad behaviors during weddings, holidays, family reunions, and gatherings in kitchens, hotels, restaurants, or industries when massive volumes of food are destroyed and wasted^1^.

In Egypt, the food waste disposal rate increased by 91 kg per person in 2021, compared to 73, 60, and 50 kg/person in 2020, 2019, and 2018, respectively. The recent in Egypt showed that wasted food costs between 100,000 and 500,000 EGP per year. According to their report, the main component of these food wastes is household starch food wastes such as bread, rice, and macaroni^2^.

One of the sustainable alternative solutions for such food waste is using microorganism-based fermentation^3^. Over the last decade, researchers have accomplished significant aims in recycling food wastes by microbial fermentation to produce biofuels, hydrogen, bioethanol, and biogas. The production of microbial α-amylase is one of the potential recycling alternatives for starch food wastes^4^.

Approximately 25% of the market for industrial enzymes is made up of α-amylase^4^. To create many products, including glucose and maltose, α-amylase, a key industrial enzyme (EC 3.2.1.1), cleaves internal 1–4 glycosidic bands of starch and other polysaccharides. One of the most popular commercial enzymes, it is a member of the GH13 (most of them), GH57, GH119, and GH1269 families. Most α-amylases are released extracellularly^5^. The submerged fermentation process was extensively utilized to produce thermostable α-amylase using thermophilic bacteria, such as *Bacillus* sp.^5^.

There are several promising future applications of the α-amylase enzyme produced from bread waste. The low-cost, sustainable production method makes α-amylase well-suited for industrial starch hydrolysis processes in textiles, paper, food, brewing, and other sectors^6^. Additionally, incorporation into detergent products as a stain remover could leverage its starch-degrading abilities for effective, eco-friendly cleaning^7^. Converting biomass sources to fermentable sugars for biofuels is another potential use of this α-amylase that warrants exploration^4^. With more research, therapeutic applications may also emerge for conditions related to abnormal starch metabolism. Further work on immobilization techniques could enhance the reusability of the enzyme^8^. Overall, the α-amylase derived from bread waste demonstrates significant versatility and this green production process creates opportunities to utilize it in various industrial, environmental, and medical contexts in the future. The promising applications span multiple fields and highlight the wide-ranging potential of this biotechnology approach to repurposing food waste^9^.

This study aims to optimize the production of α-amylase using the submerged fermentation technique by two *Bacillus* strains and the removal of starch from industrial wastewater aqueous solutions using the produced α-amylase by the BOX–Behnken design (BOX) based on response surface method (RSM). Individual and interactive effects of process variables including pH, temperature, starch concentration, enzyme concentration, and reaction time were evaluated to determine optimal conditions that maximize starch breakdown.

## Materials and methods

### Chemicals and reagents

Nutrient broth medium (OXOID CM0003B) was purchased from Oxoid, Basingstoke, England, and was used for bacteria inoculum preparation and its agar form to preserve the strains. Lugol's iodine reagent, phosphate buffer solution, HCl, calcium chloride, serum albumin (BSA), soluble starch (Sigma S-2630), and sodium alginate was purchased from Sigma, Aldrich, Germany. All chemicals were analytical grade.

### Bread food waste collection

The bread food waste was collected from local restaurants in Cairo, Egypt. It consisted of brown and white bread cuttings.

### Bread food waste analysis

The chemical analysis of bread wastes is illustrated in Table 1. Proteins, fat, carbohydrates, and fiber were determined according to AAAC and expressed as a percentage of dry matter (%).

**Table 1:** Chemical analysis of the utilized bread food wastes.

Starch wastes	Chemical analysis dry matter (%)			
	Carbohydrate	Protein	Fiber	Fat
BBW*	76.01	11.1	3.10	5.80
WBW**	69.40	9.30	2.70	3.60

*Brown bread waste (BBW), **White bread waste (WBW).

### Bacterial strains

*Bacillus amyloliquefaciens* BT 2022 (OR251122) and *B. licheniformis* Basma 87 (OP547873) strains were obtained from the Department of Agriculture Microbiology, Faculty of Agriculture, Ain Shams University, Cairo, Egypt. It was subcultured periodically using nutrient agar (NA) medium^10^ and maintained at 4 °C for further studies.

### Standard inoculum

A conical flask (250 ml) containing 50 ml of nutrient broth was inoculated with a loop of *B. amyloliquefaciens* and *B. licheniformis* cultures, individually*.* All flasks were incubated on a rotary shaker (150rpm) for 24 h at 30 °C. One milliliter of the standard inoculum contained (2.3–2.7 × 10^6^ CFU/mL)^5^.

### Preparation of bread waste for the submerged fermentation process

Bread scraps were dried for an entire night at 60 °C, then crushed and homogenized in a lab blender (Moulnix, Model: Rs383 PKR, France) using deionized water at a ratio of 1:2. Each prepared food waste sample was divided into 100 ml portions and put into conical flasks (250 ml)^7^. The pH of the flasks was adjusted to pH 7.0 using a pH meter (Hanna, Model: HI2210, UK). The liquid bread waste preparation was combined with agar 17 g/L to create a solid bread waste medium. All prepared media were sterilized for 15 min at 121 °C in an autoclave (Daihan-MaXterile, Model: Fuzzy-control 47, South Korea). Triplicates of each experiment were performed.

### Production of α amylase on solid medium

Each bacterial strain (*B. amyloliquefaciens* BT 2022 (OR251122) and *B. licheniformis* Basma 87 (OP547873) streaked on bread waste solid medium plates, which were then incubated at 30 °C for 24 h as a control. After incubation, inoculated plates were flooded with 1% Lugol's iodine reagent (10 g I_2_ and 20 g KI / L) for 20 min., then washed with distilled water to remove excess color. The ability to hydrolyze starch and the production of α-amylase were both indicated by plates with a visible halo zone^11^. The following equation ^8,9^ was used to calculate SHR (starch hydrolysis ratio):

1 $$\mathrm{HR}=\frac{\mathrm{Clear\; halo \;zone \;diameter }\;(\mathrm{mm}) }{\mathrm{Colony \;growth \;diameter }\;(\mathrm{mm})}$$

### Production of α-amylase through submerged fermentation

The standard inoculum (1 mL contained 2.4–2.7 × 10^6^ CFU/mL) of *B. amyloliquefaciens* and *B. licheniformis* cultures was added to the prepared liquid bread substrate medium and inoculated by 2%. The medium was then incubated at 30 °C for 24 hours at 150 rpm. After incubation, the growth cultures were centrifuged at 10,000 rpm for 20 min. The culture supernatants and cell pellets were harvested separately in order to evaluate enzyme activity and determine cell dry weight (CDW), respectively^13^.

### The one-factor-at-a-time (OFAT)

By experimenting with four cultural parameters (temperature: 25–40 °C, incubation time: 0–72 h, pH: 3–9, and agitation speed: 50–250 rpm), the one-factor-at-a-time (OFAT) approach was used to maximize α-amylase production. As previously mentioned, the supernatants were taken after each optimization step and tested for enzyme activity and bacterial growth^14^.

#### Estimation of α- amylase activity

The activity of the enzyme α-amylase was measured using the iodine method^15^. Briefly, In 100 ml of distilled water, one gram of soluble starch (Sigma S-2630) was gelatinized for 15 min at 100 °C. One milliliter of the crude enzyme was combined with 0.5 ml of 0.1 M phosphate buffer solution (PBS), pH 7.0, and one milliliter of the gelatinized starch solution. The reaction mixture was incubated at 60 °C for 30 min. Following the addition of 1 ml of the iodine reagent (5 mM I_2_ and 5 mM KI), 1 ml of 1M HCl was used to stop the reaction. Enzyme activity was determined by measuring Optical density (OD) at 620 nm. versus control treatment without substrate (starch) addition. The disappearance of 1 mg of the iodine-binding starch mixture per minute is the definition of one unit (U/mL) of α-amylase in the starch-iodine assay. The following formula was used to calculate the enzyme activity (U/mL)^10^.

2 $$\varvec{\upalpha } - {\mathbf{amylase}}\;{\mathbf{activity}}\;({\mathbf{U}}/{\mathbf{mL}}) = \frac{{{\text{A}}620{\text{nm}}\,{\text{control}} - A620{\text{nm}}\;{\text{sample}}}}{{{\text{A}}620{\text{nm}}/{\text{mg}}\,{\text{starch}}}} \times \frac{1}{{{\text{A}}620{\text{nm}}/{\text{mg}}\,{\text{starch}} \times 30 \times {\text{dilution}}\;{\text{factor}}}}$$

where A_620_ nm control is the absorbance of the starch without active enzyme, A_620_ nm sample is the absorbance for the starch with active enzyme, and A_620_ nm/mg starch is the absorbance for 1 mg of starch as obtained from the starch standard curve.

Using the following equations^6^, the effective yield of α-amylase production was determined.

3 $$\mathrm{Specific\; enzyme \;yield }=\mathrm{ Enzyme\; produced }\;(\mathrm{U}/\mathrm{mL }) /\mathrm{ cell \;mass\; formation }\;(\mathrm{g}/\mathrm{L})$$

#### Determination of total protein

The measurement of protein was done^16^, using Biuret reagent and bovine serum albumin (BSA) as standard for enzyme-specific activity estimation. For biuret reagent preparation, 300 ml of 10% (w/v) NaOH was added with stirring, to 500 ml of a solution containing 0.3% copper sulfate pentahydrate and 1.2% sodium potassium tartrate, then diluted to one liter. One gram of potassium iodide was added per liter and stored in the dark. It was used for protein determination. The enzyme-specific activity using the following equations ^17^

4 $$\mathrm{Specific\; activity }\;(\mathrm{U}/\mathrm{mg\; protein}) =\mathrm{ Enzyme\; activity}/\mathrm{protein \;content}$$

#### Crude α-amylase immobilization

For α-amylase crude enzyme immobilization, calcium alginate beads were prepared by adding an equal volume of crude enzyme and sodium alginate (2% w/v) solution, then transferring the mixture to a syringe (0.8 mm diameter), and beads were formed by dropping the mixture into CaCl_2_ (5% w/v) solution with gentle stirring at cool (4 °C) for 1 h. The crude enzyme beads were extracted via filtration and then thoroughly washed with distilled water to eliminate excess CaCl_2_ and residual α-amylase. Before usage, the beads were dried with filter paper (Whatman no. 1) and exposed to open air for 1 h. The CaCl_2_ filtering residues washing solution was collected to evaluate enzyme activity, and the immobilization yield was determined^17^.

#### Reusability and immobilization efficiency of immobilized crude α-amylase

α-amylase immobilized on calcium alginate beads was reused and tested several times to estimate starch hydrolysis efficiency and stability. A solution of (1:1 v/v) 1% gelatinized starch and pH 9.0 was used to estimate the enzyme reusability, after each 10-min run, the beads were separated, cleaned three times with distilled water, and stored at 4 °C until the next run. For each batch run, a new fresh reaction mixture was added, and the enzyme activity was estimated as previously mentioned. The control activity of the tested beads for each run was compared to the first run (100% activity)^17^. The immobilization yield, the immobilized enzyme activity in calcium alginate beads, was determined using the following equation ^17^

5 $$\mathrm{Immobilization\; efficiency }(\mathrm{IF})\;\mathrm{\% }= (\mathrm{Activity\; of \;immobilized \;enzyme }/\mathrm{ A}-\mathrm{B}) \times 100$$

where A is the activity of the free enzyme added, and B is the activity of a remaining enzyme in washed water and filtered calcium chloride solution. Both A and B were evaluated from the amount of reducing sugars produced enzymatically in the corresponding solutions.

#### Immobilized Crude α-amylase enzyme application as a desizing agent

Desizing in textile manufacturing is defined as the percentage of starch removal from the treated clothes with starch in the starching step. To imitate the desizing process in textile manufacture, a 55-inch piece of starch-saturated fabric is employed. Various concentrations of crude α-amylase were prepared using PBS pH 9.0 with a two-fold dilution technique as the concentration of a solution by a factor of two that reduced the original concentration by one-half to form concentrations of 695.2, 347.6, 173.8, 86.9, 43.4, 21.7, and 10.9 U/mL. The prepared concentrations of the enzyme were immobilized using calcium alginate, as previously mentioned. Followed by soaking the cloth strips in 100 ml of different immobilized crude α-amylase concentrations for 15 min and pH 9.0. The treated cloth strips were placed in an incubator at the optimum temperature for the enzyme reaction. The cloth strips were washed under running water and dried in the oven at 60 °C for 1 h. The cloth strips were weighed before and after drying to evaluate the desizing efficiency. The following formula is used to calculate desizing efficiency^15^.

6 $$\mathrm{\%Desizing}= (\mathrm{Weight\; of \;cloth \;fabric \;saturated \;with \;starch \;before \;desizing }-\mathrm{Weight \;of \;cloth \;fabric \;starch \;after \;desizing})/\mathrm{Weight \;of \;cloth \;fabric \;saturated \;with \;starch \;before \;desizing}) \times 100$$

#### BOX–Behnken design (BBD) for the optimization of starch hydrolysis using *Bacillus* strain

Table 2 discusses the use of response surface methodology (RSM) and Box–Behnken design (BBD) for the optimization of a starch hydrolysis process^18^. Five factors were considered as independent variables: pH, temperature, starch concentration, α-amylase concentration, and reaction time. Starch hydrolysis percentage was used as the dependent variable or response. A total of 46 experiments were conducted using BBD based on RSM to optimize the five independent variables. The objective was to determine the optimal levels of the variables to achieve maximum starch hydrolysis percentage. The experimental range and levels of the independent variables used in the study were selected. In summary, RSM and BBD can be applied to optimize a starch hydrolysis process by investigating the effects of various reaction parameters on the degree of starch hydrolysis, Table 2. The fitting on the efficiency of eliminating color was implemented using the determination coefficient (R^2^) and the adjusted R^2^. A mathematical model^19^ was developed to correlate the response surface using a quadratic equation. Analysis of variance (ANOVA) and the coefficient of determination (R^2^) were used to evaluate the significance of the regression model. The model terms were assessed based on their p-values at the 95% confidence level using response surface regression analysis software generating surface plots and contour plots of the response models.

**Table 2:** Tested ranges and levels for the independent process factors.

Factor	Name	Units	Minimum	Maximum
A	pH		6.00	9.00
B	Temperature	°C	30.00	60.00
C	Starch	%	70.00	100.00
D	Enzyme	%	5.00	50.00

#### Statistical analysis

The obtained data were analyzed statistically using the SPSS Statistics (version 19) software**,** The Tukey test was estimated at a *p*-value of ≤ 0.05^18^.

#### Ethical Statement

This article does not contain any studies with human participants or animals performed by any of the authors.

## Results

### Qualitative screening of starch waste degradation and enzyme production using the starch hydrolysis ratio (SHR) test

**Figure 1:**
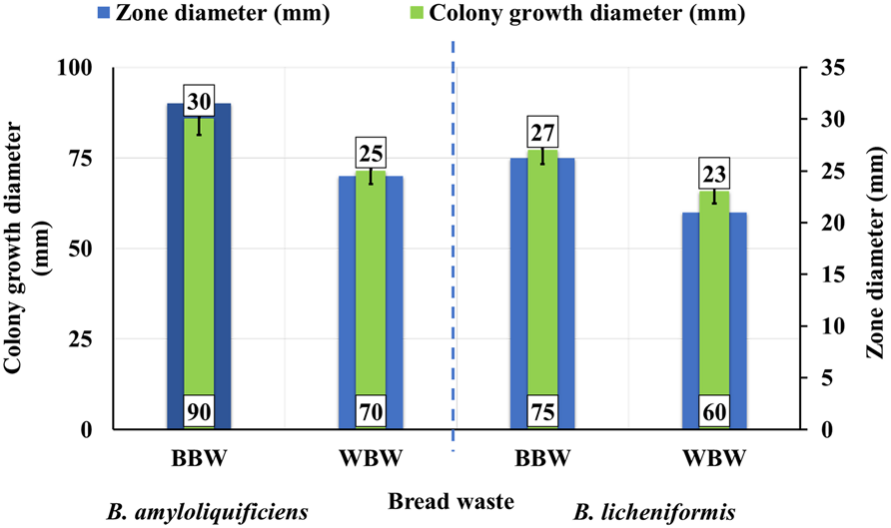
Qualitative screening of bread food waste degradation using the starch hydrolysis ratio (SHR) test after incubation for 24 h at 30 °C whereas; Brown bread waste (BBW), and White bread waste (WBW).

As reviewed in Fig. 1, The most efficient strain was *B. amyloliquefaciens,* which exhibited the largest halo zones of 90, and 70 mm and colony diameters of 30, and 25 mm for BBW, and WBW, respectively. Compared to *B. licheniformis* with the lowest starch clear halo zone of 75, and 66 mm and cell growth diameter of 27, and 23 mm for BBW, and WBW, respectively. The superiority of SHR formation was scored for *B. amyloliquefaciens* with 3.0, and 2.8, 2. while it was the lowest for *B. licheniformis* with 2.8, and 2.6 for BBW, and WBW, respectively.

### The α-amylase fermentation process and quantitative estimation

**Figure 2:**
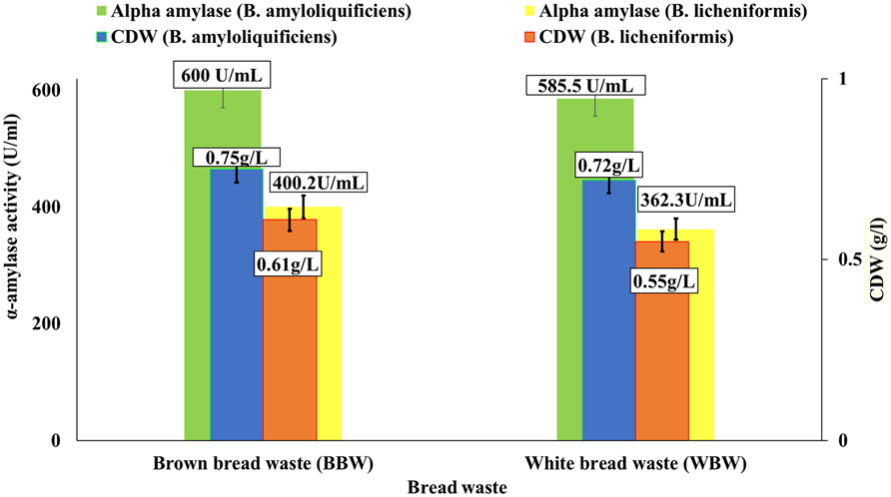
Quantitative *B. amyloliquefaciens* and *B. licheniformis* α-amylase production using different bread wastes after 24 h at 30 °C of fermentation under submerged conditions (150rpm). whereas; Brown bread waste (BBW), and White bread waste (WBW).

As demonstrated in Fig. 2, the best bread waste was BBW with *B. amyloliquefaciens* for the α-amylase production and starch degradation reached 600 U/mL. Moreover, utilizing Brown bread waste boosts cell growth and α-amylase production, as the cell growth increases, the enzyme production increases too. Also, the correlation coefficient among them was very strong, recording an R-value of 0.9818 and 0.9757 for *B. amyloliquefaciens* and *B. licheniformis*, respectively, for all testes starch food wastes.

### Optimization of α-amylase fermentation temperature

**Figure 3:**
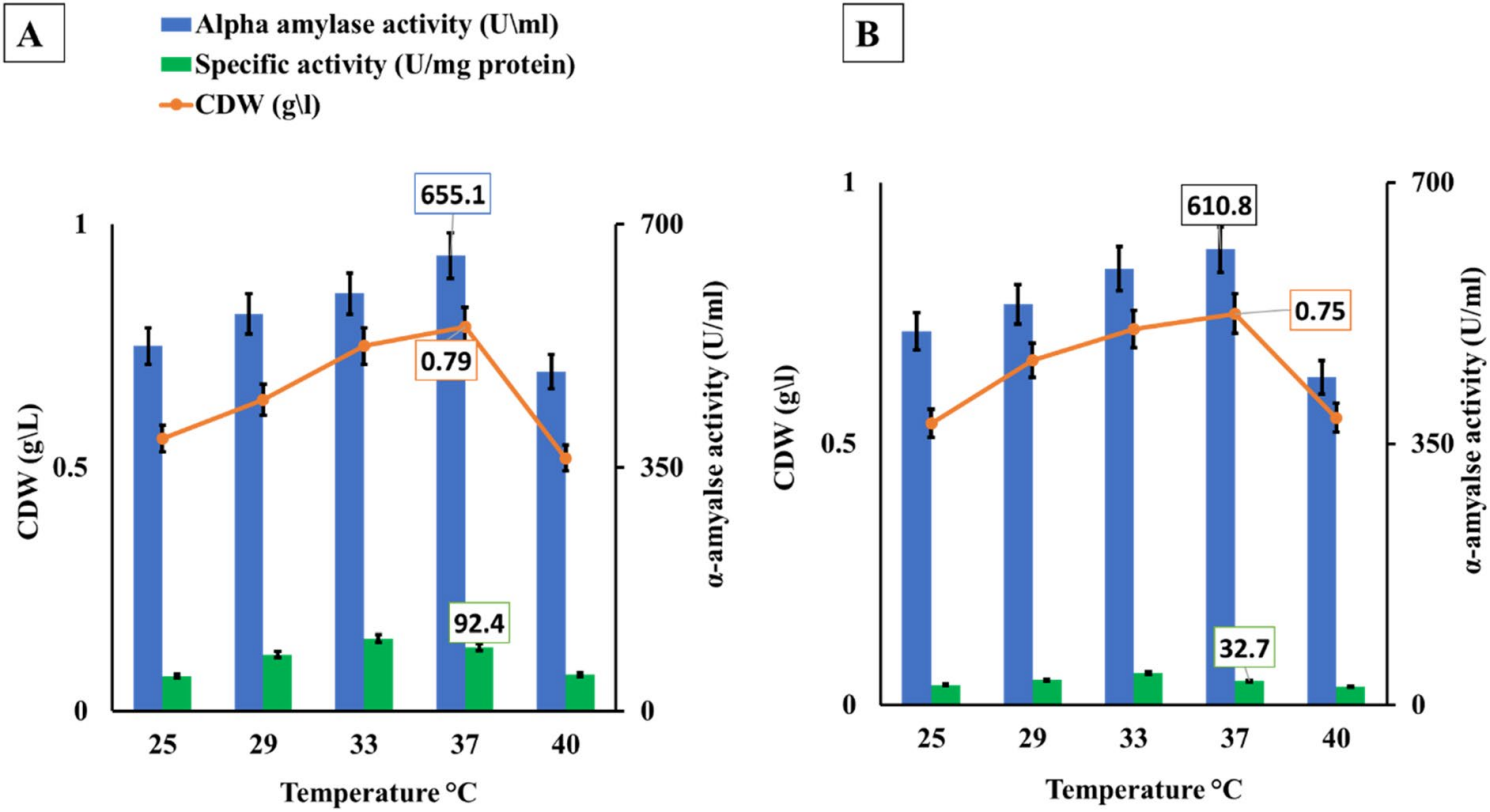
Temperature optimization for α-amylase and cell mass production by *B. amyloliquefaciens* using different bread food wastes after 24 h of fermentation and under submerged conditions(150rpm). Whereas; A = Brown bread waste (BBW), and B = White bread waste (WBW).

The optimum temperature for α-amylase fermentation and cell growth was determined at various temperatures ranging from 25 to 40 °C. It was obvious from Fig. 3 that *B. amyloliquefaciens* produced the most α-amylase (655 U/mL) when grown in BBW at 37 °C. While it decreased by 10% and reached 610.8 U/mL when WBW was used. The lowest α-amylase activity was found at 25 and 50 °C for all studied starch wastes. The activity of α-amylase with bread wastes increased at 37 °C by 1.09, and 1.05 times, respectively compared with the control temperature of 30 °C, Fig. 3. Also, the correlation between α-amylase and temperature was very strong, recording an r-value of 0.873, and 0.765 for BBW and WBW, respectively. The specific activity was estimated at 50.9, 81.8, 104.7, 92.4, and 52.5 U/mg protein with BBW while it was 27.09, 33.8, 24.8, 32.7, and 24.6 U/mg protein with WBW for 25, 29, 33, 37, and 40 °C incubation temperature, respectively.

### Optimization of α-amylase fermentation pH

**Figure 4:**
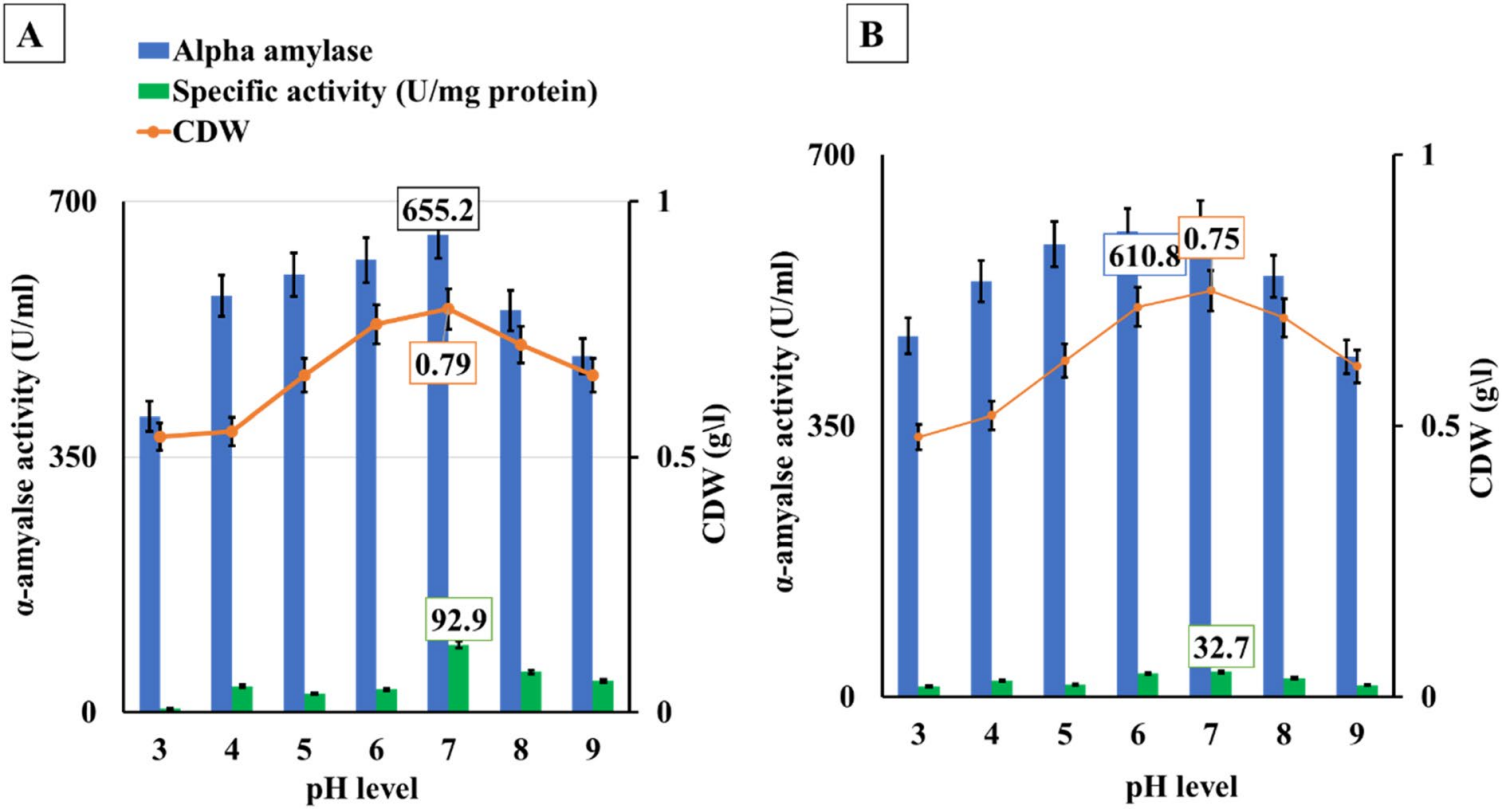
pH optimization for *B. amyloliquefaciens* for α-amylase production using different bread food wastes at 37 °C for 24 h and under submerged conditions(150rpm). Whereas; A = Brown bread waste (BBW), and B = White bread waste (WBW).

According to data presented in Fig. 4, α-amylase production peaked at pH 7.0 (655.3 U/mL) using BBW at 37 °C, and any further rise or drop in fermentation pH level over 7.0 resulted in a decrease in α-amylase and cell mass production. When the pH of BBW and WBW was elevated from pH 7.0 to pH 9.0, the amylase activity decreased by 25.5% and 28.03%, respectively. Subsequently, the cell mass decreased by 16.5% and 18.7%, respectively. The correlation between α-amylase and temperature was very strong, recording R values of 0.873, and 0.765 for BBW, and WBW respectively. It was found that amylase activity was lowered by 38.2% and 23.6% when the pH was decreased from pH 7.0 to pH 3.0, respectively. The specific activity was 5.2, 36.1, 25.6, 31.8, 92.9, 55.6, and 43.5 U/mg protein with BBW while it was 13.9, 20.8, 15.9, 30.7, 32.7, 24.3, and 15.4 U/mg protein with WBW under pH point of 3, 4, 5, 6, 7, 8, and 9.

### Optimization of α-amylase fermentation time

**Figure 5:**
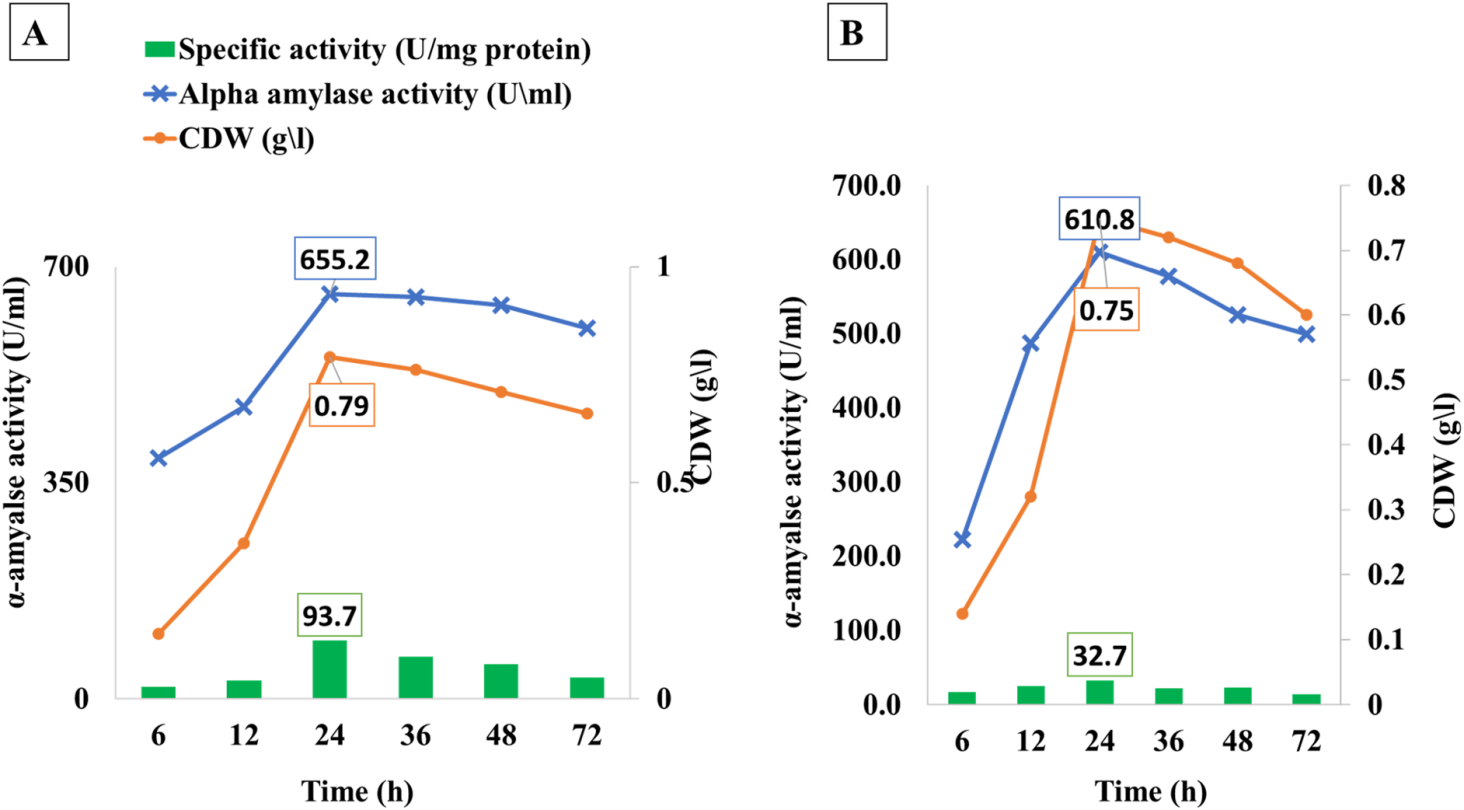
Fermentation time optimization of *B. amyloliquefaciens* for α-amylase production using different bread food wastes at 37 °C after 24 h under submerged conditions(150rpm). Whereas; A = Brown bread waste (BBW), and B = White bread waste (WBW).

The data in Fig. 5 showed that the highest amylase production occurred during the first 24 h of fermentation and any further incubation didn't exhibit any α-amylase production increase with BBW and WBW. The enzyme activity of the BBW and WBW dropped by 0.8% and 5.4%, respectively, after 48 h of the fermentation period. Also, after 72 h of incubation, the enzyme production was minimized by 2.7% & 13.9%, and 8.4% for BBW, and WBW, respectively. The correlation between α-amylase and fermentation time was very strong, recording r values of 0.928 and 0.905 for BBW, and WBW, respectively. The relative activity was 19.9, 289, 93.7, 67.7, 55.4, and 34.1 U/mg protein for BBW and 17.8, 25.7, 32.7, 22.3, 23.04, and 14.7 U/mg protein for WBW for an incubation time of 6, 12, 24, 36, 48, 72 h, respectively.

### Optimization of α-amylase production shaking-speed

**Figure 6:**
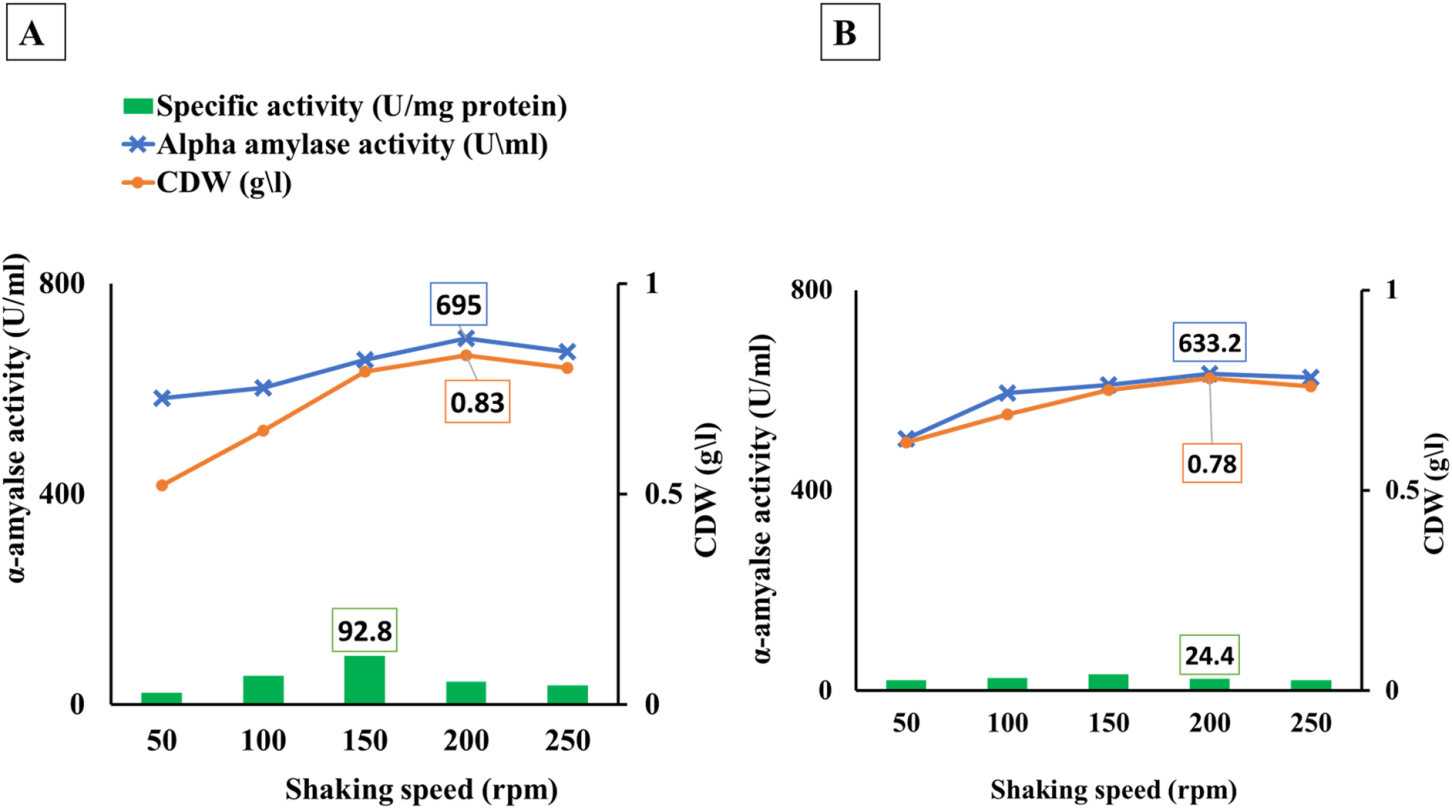
Shaking speed optimization of *B. amyloliquefaciens* for α-amylase production using different starchy wastes at 37 °C after 24 h under submerged conditions (150rpm). Whereas; A = Brown bread waste (BBW), and B = White bread waste (WBW).

The ideal shaking speed at 200 rpm has risen by 6.1 and 4.6% for BBW and WBW, respectively, according to Fig. 6. While, at 250rpm α-amylase production increased only by 2.3 and 2.2% for BBW and WBW, respectively. The correlation between α-amylase and fermentation shaking speed was very strong, recording r values of 0.893 and 0.962 for BBW and WBW, respectively. The relative activity was 22.5, 54.2, 92.8, 43.7, and 36.2 U/mg protein for BBW and 20.7, 25.9, 32.5, 24.4, and 21.8 U/mg protein for WBW for shaking speed of 50, 100, 150, 200, and 250rpm, respectively.

It could be concluded that α-amylase production and cell growth were optimal with *B. amyloliquefaciens* at a temperature of 37 °C, a pH level of 7.0, an agitation speed of 200 rpm, and a fermentation time of 24 h.

### α-amylases crude enzyme immobilization on Ca-alginate

**Figure 7:**
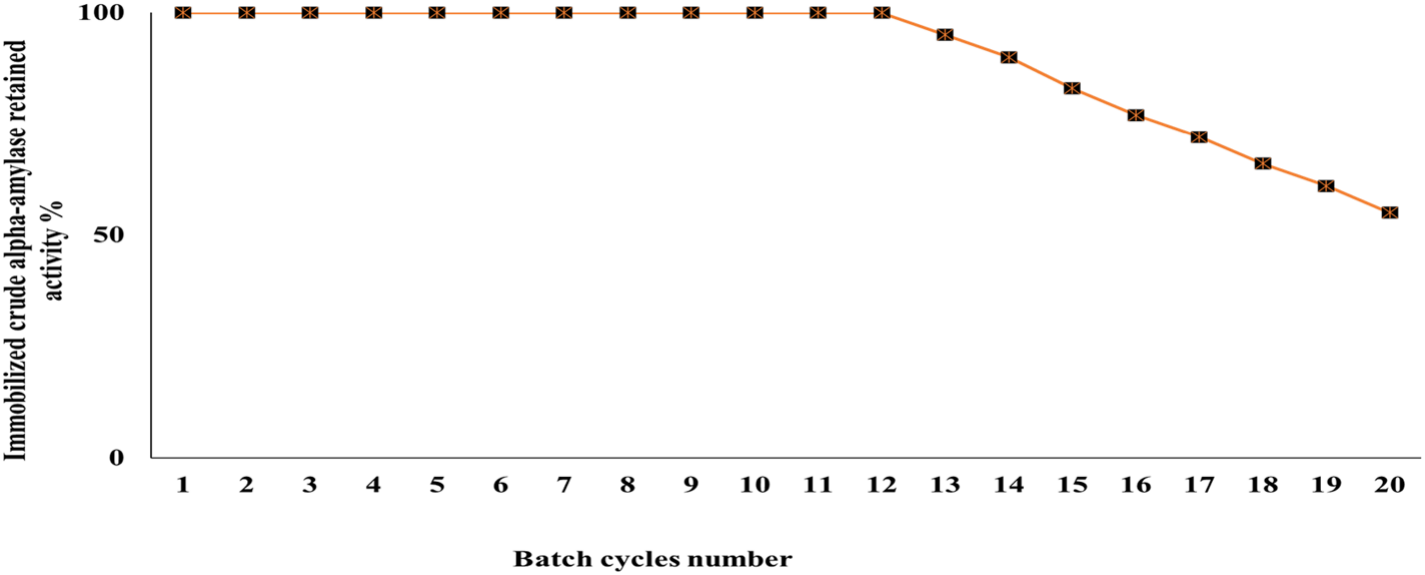
Reusability of *B. amyloliquefaciens* immobilized crude α-amylase on Ca-alginate.

The immobilization of *B. amyloliquefaciens* crude α-amylase in Ca-alginate polymer was investigated. The immobilization efficiency and activity yield of crude immobilized enzyme were estimated by Free units (FU) of 695.2 U/mL, Remaining units in solution (RUS) after immobilization was 60 U/mL, and the Immobilized units (IU) on the calcium alginate beads were estimated by 615 U/mL. From the previous records, the activity yield (AY) was calculated to be 88.5%, and the immobilization efficiency (IE) was 96.9%. After the first batch of the hydrolysis process for the immobilized α-amylase crude enzyme, it displayed a repeated operating batch of immobilized crude α-amylase stability curves as shown in Fig. 7. The encapsulated enzyme's activity was roughly 100% preserved over the first twelve batch cycles, according to the results of 20 repeated batches.

### BOX–Behnken *design* for optimizing wastewater treatment

As shown in Table 3, the study found that the quadratic model developed was statistically significant for starch decolorization. This is shown by the *p*-value being less than 0.05, showing that the factors had a significant effect. The F value of 6.51 and Prob > F value less than 0.0500 show that the model is significant for starch hydrolysis. The low Prob > F value (less than 0.05) with a coefficient of determination (R^2^ = 0.83) shows that the model terms had a significant effect on the response. D, E, and E^2^ were the most significant factors. In summary, the factors of pH, temperature, time, starch concentration, and α-amylase were found to have a significant impact on starch decolorization according to the mathematical model developed in the study. The mathematical expression for the relationship between starch degradation with the independent variables: pH, temperature, time, starch concentration, and α-amylase is shown in terms of coded factors in Eq. (6): Coded equation.

**Table 3:** Experimental design matrix with the experimental and predicted values for starch hydrolysis efficiency (%) using α-amylase from *B. amyloliquefaciens.*

Run No	pH	Temp. (°C)	Starch (%)	Enzyme conc. (%)	Contact time (min.)	Experimented Starch hydrolysis (%)	Predicted starch hydrolysis (%)
1	9.0	45	70	27.5	15	98.67	99.74
2	7.5	60	85	5.00	15	97.53	90.24
3	7.5	60	85	50.0	15	98.93	90.05
4	6.0	45	70	27.5	15	85.60	89.82
5	7.5	45	85	27.5	15	85.67	86.43
6	7.5	45	85	27.5	15	95.73	86.43
7	6.0	30	85	27.5	15	92.67	93.62
8	7.5	60	70	27.5	15	98.80	97.54
9	7.5	30	85	5.00	15	86.27	83.39
10	7.5	60	85	27.5	30	95.60	101.2
11	7.5	45	85	50.0	30	94.27	99.11
12	7.5	45	70	27.5	30	97.31	87.85
13	7.5	45	85	27.5	15	94.53	86.43
14	7.5	45	85	27.5	15	88.67	86.43
15	7.5	30	85	50.0	15	98.13	93.68
16	6.0	45	85	27.5	30	98.01	88.48
17	7.5	60	85	27.5	0	34.00	47.64
18	7.5	45	85	27.5	15	67.33	86.43
19	9.0	60	85	27.5	15	98.80	98.36
20	7.5	45	85	27.5	15	86.67	86.43
21	6.0	60	85	27.5	15	97.00	96.50
22	6.0	45	100	27.5	15	95.78	95.61
23	7.5	45	85	5.00	30	57.13	69.43
24	7.5	30	85	27.5	30	86.00	86.53
25	7.5	45	70	50.0	15	72.87	88.86
26	7.5	45	100	27.5	30	95.93	95.17
27	6.0	45	85	50.0	15	84.67	83.67
28	7.5	60	100	27.5	15	98.07	97.23
29	7.5	45	70	5.00	15	92.33	86.14
30	9.0	45	85	27.5	0	51.20	51.11
31	6.0	45	85	5.00	15	75.13	87.28
32	7.5	45	100	50.0	15	78.93	90.19
33	7.5	45	85	50.0	0	47.80	33.98
34	7.5	30	85	27.5	0	50.47	59.07
35	7.5	45	70	27.5	0	57.93	55.67
36	9.0	45	85	27.5	30	98.13	94.63
37	6.0	45	85	27.5	0	57.13	51.01
38	7.5	30	70	27.5	15	98.73	96.62
39	9.0	45	85	50.0	15	99.40	95.46
40	7.5	45	100	27.5	0	39.93	46.36
41	7.5	30	100	27.5	15	96.60	94.93
42	9.0	30	85	27.5	15	97.00	98.01
43	7.5	45	85	5.00	0	59.93	53.56
44	9.0	45	85	5.00	15	72.53	81.74
45	9.0	45	100	27.5	15	95.27	91.95
46	7.5	45	100	5.00	15	93.73	82.81

ANOVA analysis							
Model	*P* Value				C.V	R^2^	Adjusted R^2^
	Model	E	DE	E^2^			
Significant	< 0.0001	< 0.0001	0.0207	< 0.0001	11.94	0.8389	0.7099

$$\begin{aligned} Y = & 86.4333 + 1.56312*A + 0.804375*B + - 0.499375*C \\ + & 2.52521*D + 20.2492*E + - 0.633333*AB + - 3.395*AC \\ + & 4.3325*AD + 1.51333*AE + 0.349167*BC + - 2.61667*BD \\ + & 6.51667*BE + 1.16667*CD + 4.15667*CE + 12.3167*DE \\ + & 3.94444*A^{2} + 6.245*B^{2} + 3.90222*C^{2} + - 3.33833*D^{2} + - 19.0725*E^{2} . \\ \end{aligned}$$

**Figure 8:**
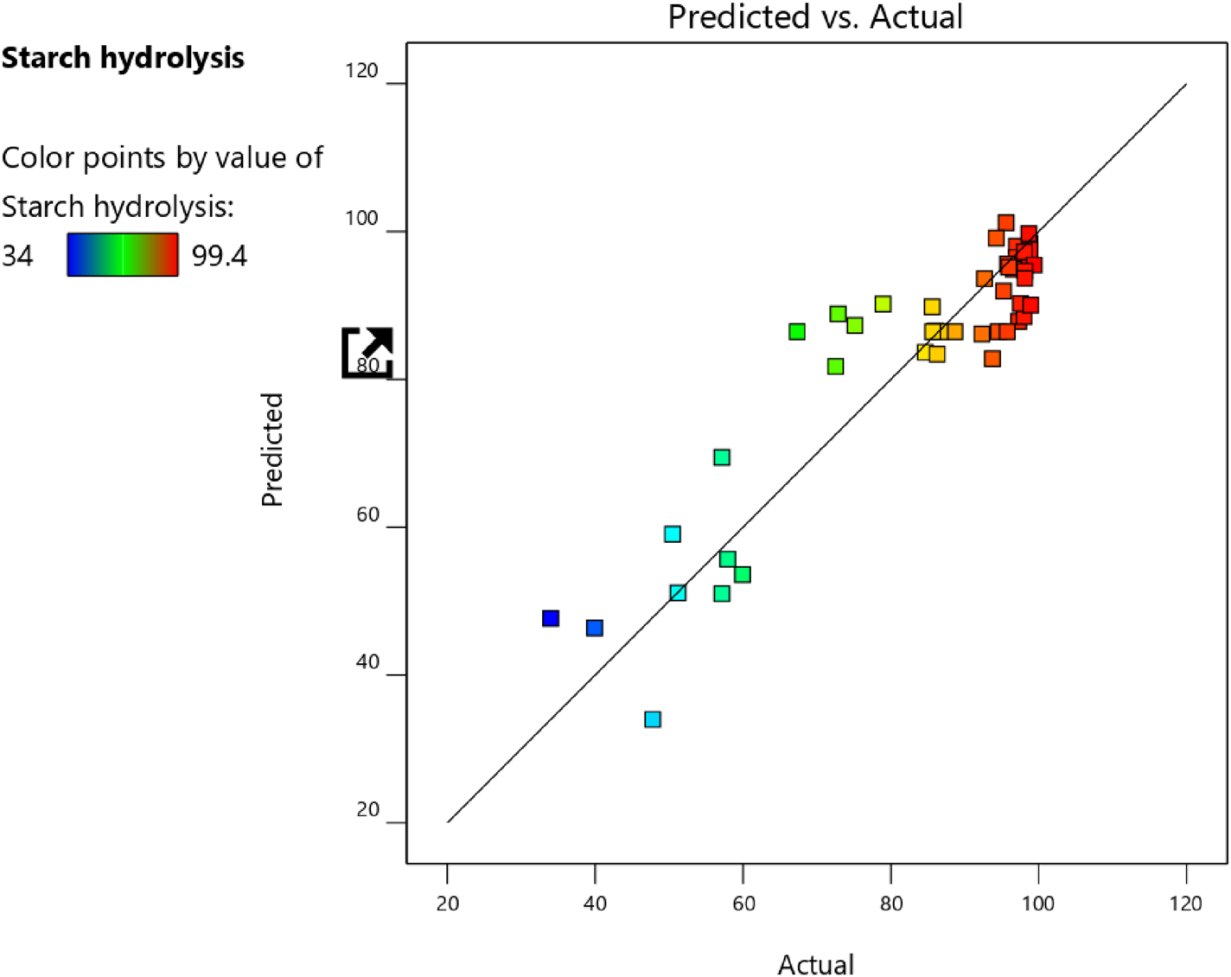
Plot of the predicted values versus the observed values of starch hydrolysis efficiency (%) using α-amylase from *B. amyloliquefaciens.*

From Fig. 8, it was also confirmed that the experimental (actual) values were very close to the predicted values.

**Figure 9:**
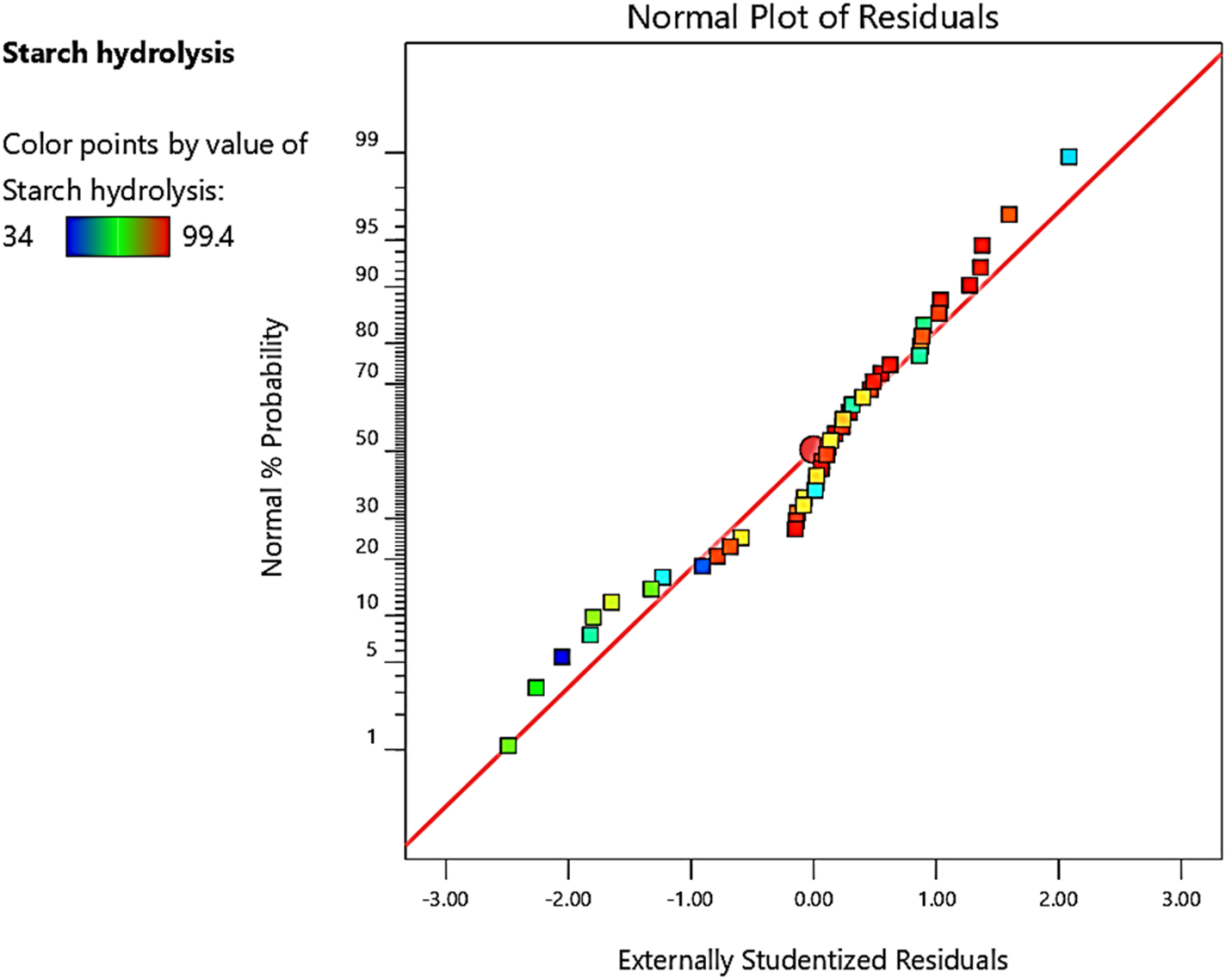
The studentized residuals and normal percentage of probability residuals for starch hydrolysis starch hydrolysis efficiency (%) using α-amylase from *B. amyloliquefaciens.*

The ANOVA results showed that the quadratic model (Eq. 6) is adequate for predicting starch hydrolysis in the variables’ studied range. A normal probability plot is used to check the normality distribution of the residuals. Great deviation from normality was not observed in the normal probability plots of the residuals (Fig. 9).

### Response surface plots

**Figure 10:**
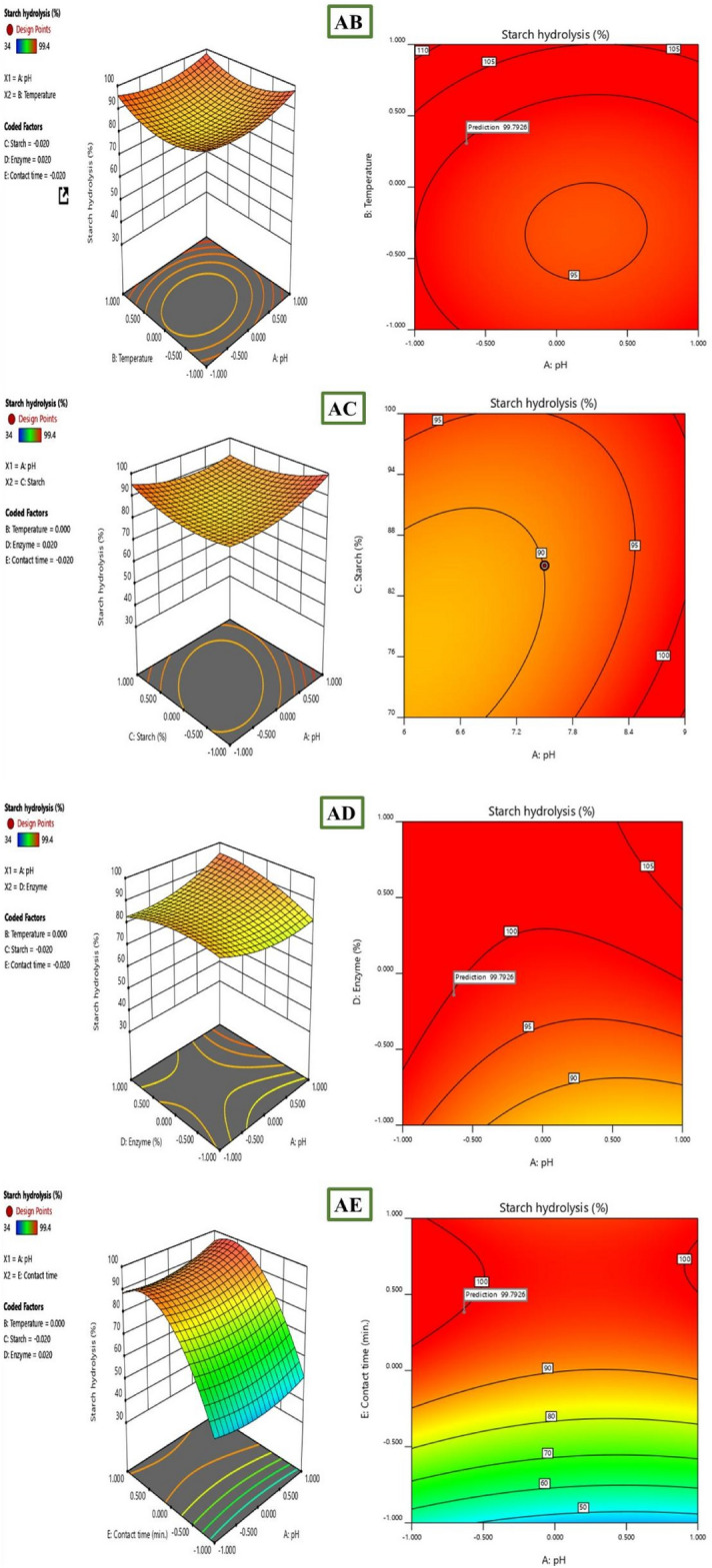

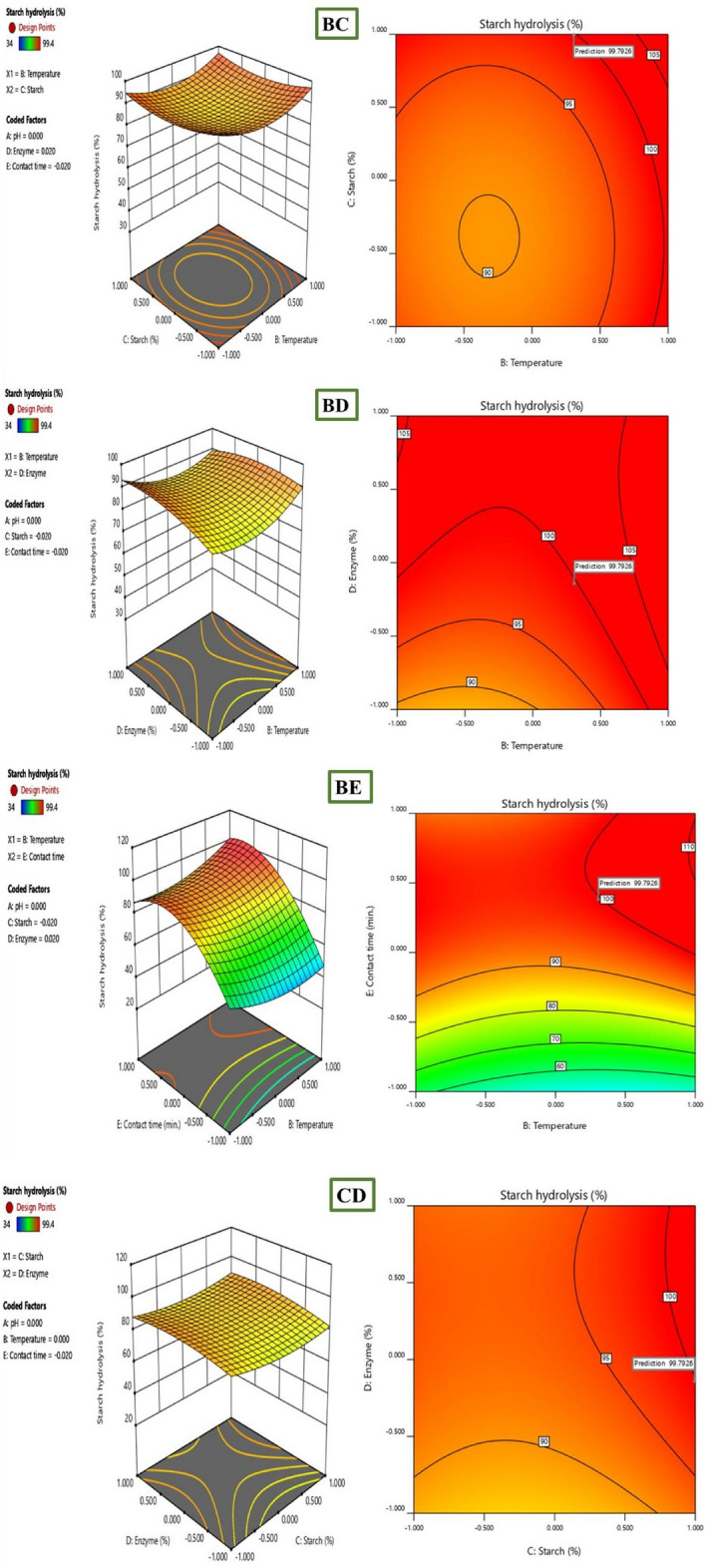

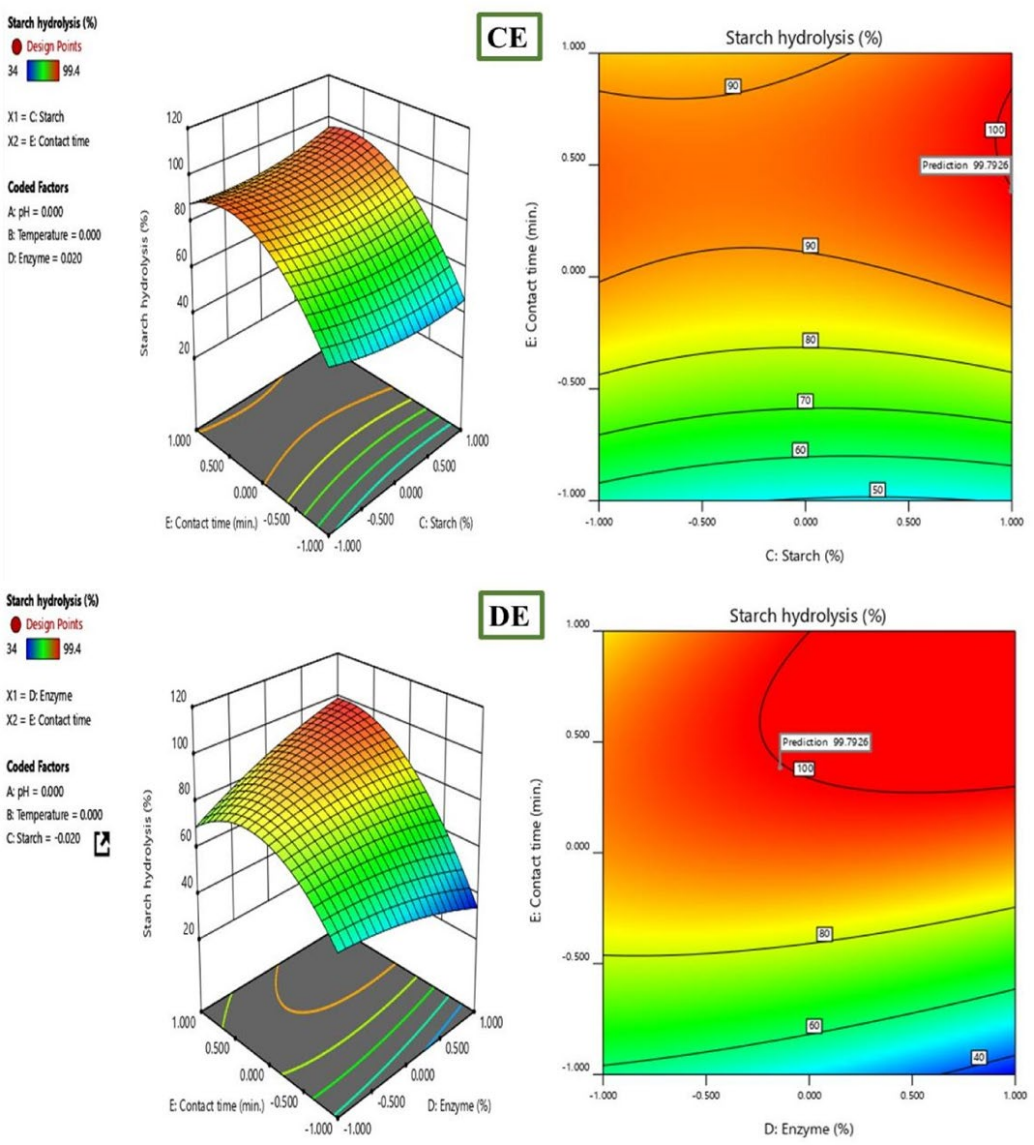
3D surface and 2D contour plot for the interactive factors of starch hydrolysis starch hydrolysis efficiency (%) using α-amylase from *B. amyloliquefaciens.*

**Figure 11:**
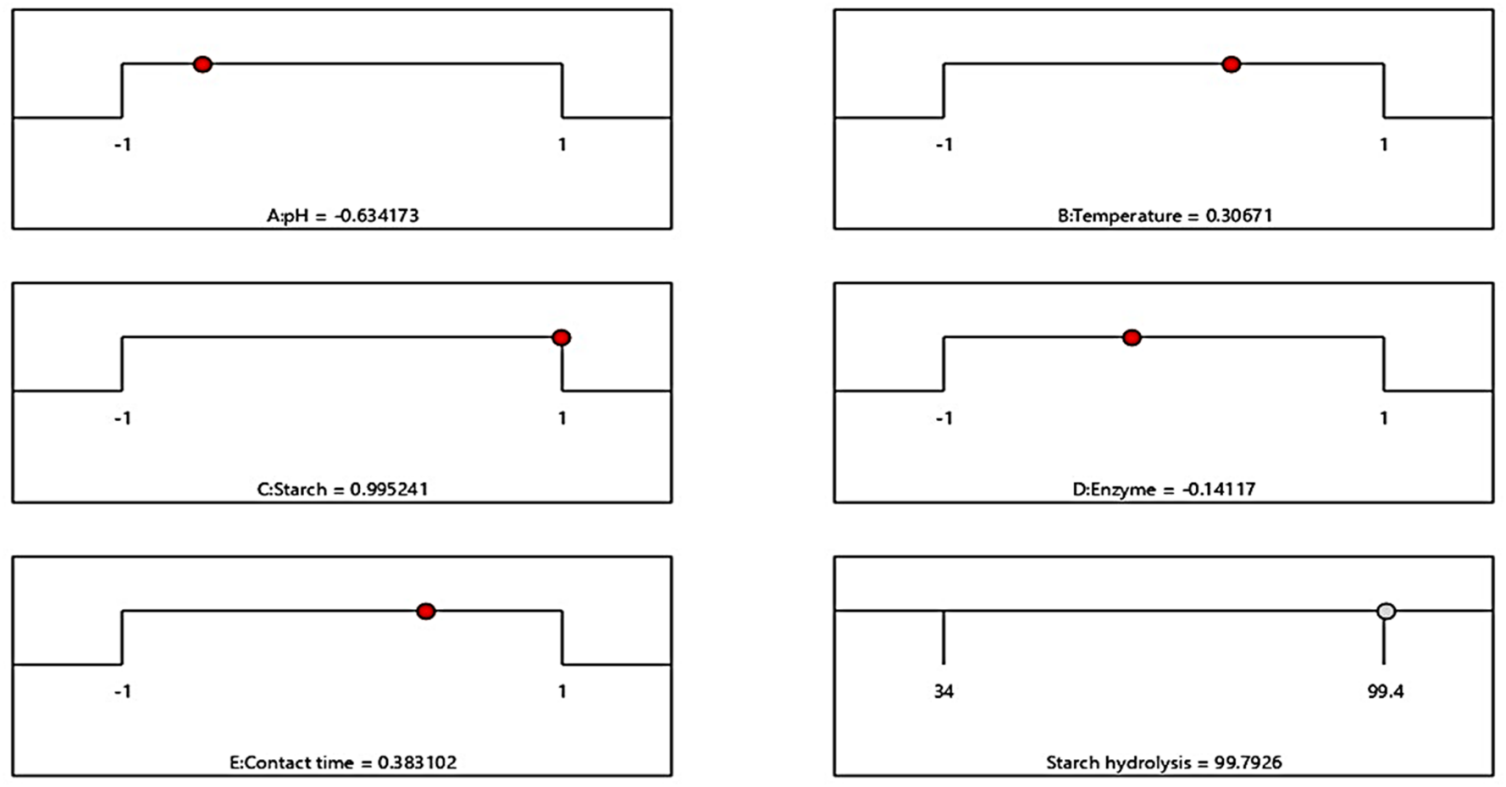
The desirability effect for starch hydrolysis efficiency (%) using α-amylase from *B. amyloliquefaciens.*

**Figure 12:**
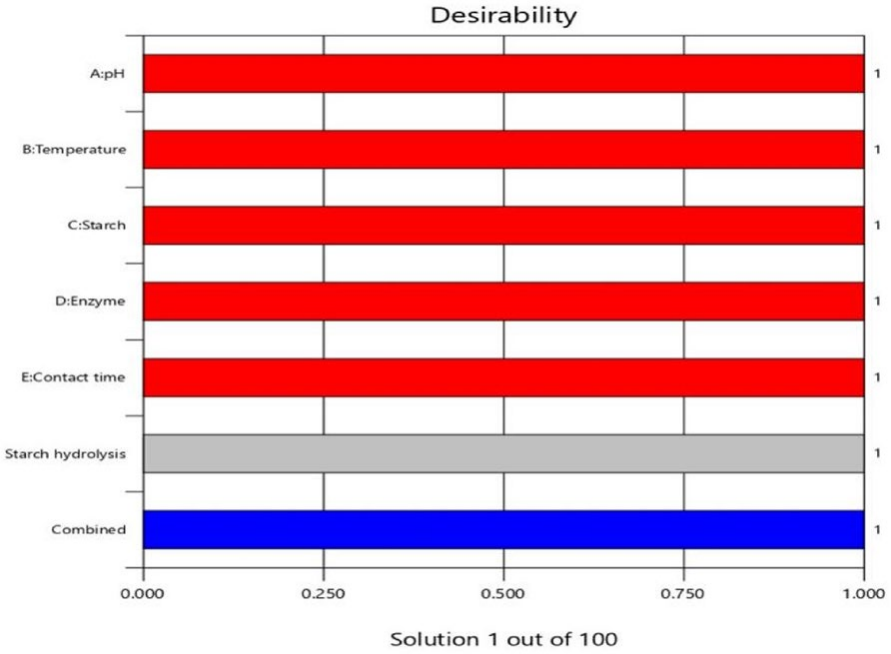
Desirability effect of the individual operating parameter for starch hydrolysis efficiency (%) using α-amylase from *B. amyloliquefaciens.*

Further investigation into the interactive effects of process variables on starch decolorization using advanced process dye involved the creation of three-dimensional (3D) surface curves and two-dimensional (2D) figures. These plots displayed the relationship between two independent variables while holding the other variables at their central level. The figures depicted the response, which was the percentage hydrolysis resulting from the interactions between the variables. In Fig. 10, it was observed that time played a crucial role in starch hydrolysis. Increased contact time led to a higher amount of starch being hydrolyzed because of the availability of active sites. However, the concentration of starch and enzyme over time hindered starch hydrolysis as they blocked the active sites. To determine the optimal conditions for starch hydrolysis, the Design Expert software (Stat-Ease, 12 trial version) was used for optimization. The software determined that the optimum conditions were a pH of 9, a temperature of 45 °C, a starch concentration of 70%, and an enzyme concentration of 27 mL, and a reaction time of 15 min. Under these conditions, the predicted efficiency of hydrolysis was 99. 4%. To validate this prediction, an experiment was conducted, resulting in an experimental value of 98. 63%, which closely aligned with the predicted percentage of hydrolysis (99. 4%). The desirability of a model is determined by its proximity to unity. Here, the desirability was 1, as shown in Figs. 11 and 12. This confirms the applicability of the model and the predicted responses. The desirability values for the individual process variables were also close to unity, showing that each operating parameter satisfied the model. These values further showed how well each variable aligned with the model.

#### The desizing process using crude α-amylase

**Figure 13:**
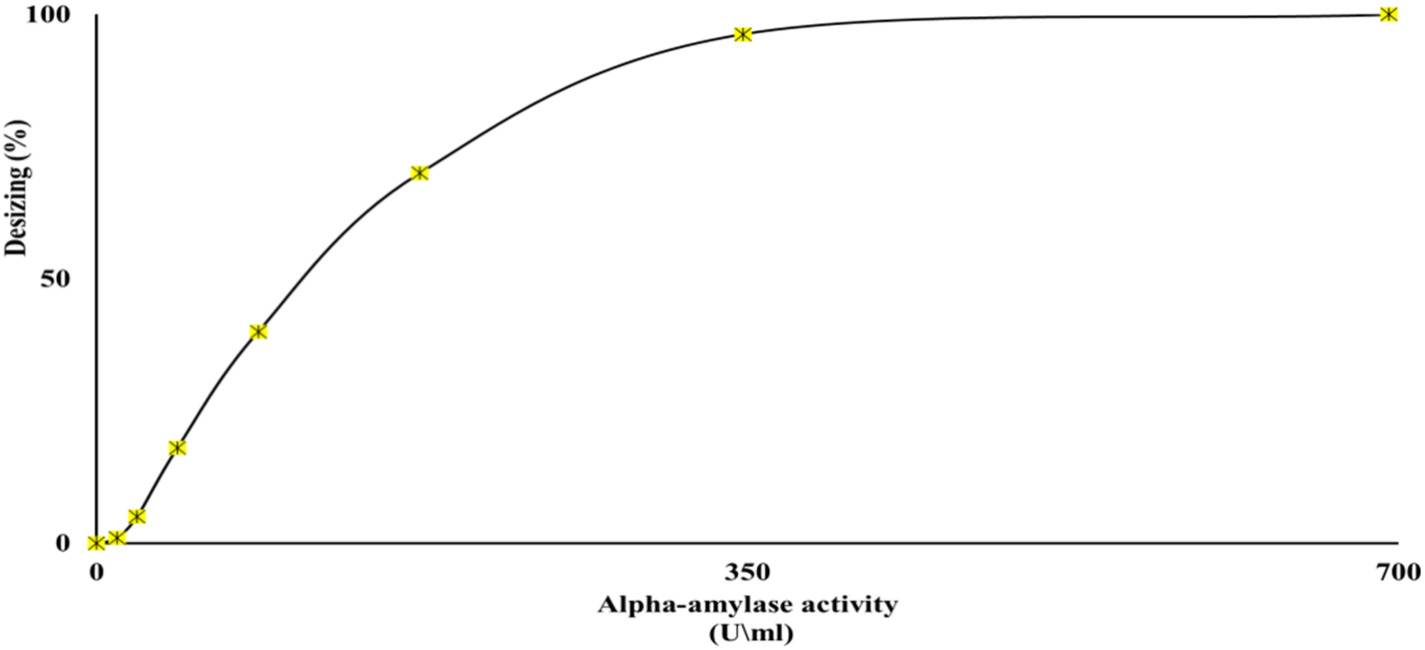
Desizing efficiency of *B. amyloliquefaciens* crude α-amylase at 45 °C, and pH 9.0 for 15 min.

The desizing of the cloth strips was impacted by crude-α amylase different concentrations of 10.9, 21.7, 43.4, 86.9, 173.8, 347.5, and 695 U/mL. Data clearly showed that a concentration of 347.5 U/mL gave the greatest desizing efficiency of 96.3% as presented in Fig. 13. On the other hand, any additional increases in enzyme concentration had no significant effect on fabric desizing.

## Discussion

*Bacillus* species are often the predominant bacteria responsible for degrading most food wastes, especially those high in starch content^19^. Also, *B. amyloliquefaciens*, *B. subtilis*, *B. licheniformis*, and *B. polymyxa* have been linked to food waste management, biodegradation, and α-amylase production^3^. Our results proved that natural starch food wastes are a good source for α-amylase production. So, natural starch sources as wheat, potatoes, and rice, were found to be appropriate substrates for α-amylase production by most of the *Bacillus* bacterial species^21^. Also, The superiority of α-amylase synthesis and cell growth with BBW way be attributed to the presence of several amino acids in bread such as biotin, nicotine, thiamine, and pantothenic acid, which enhance microbial growth and α-amylase secretion^22^. In contrast to prior studies, many utilized raw materials and wastes need an enzymatic pretreatment with amylases, proteases, and lipases to be utilized as commercial fermentation media. Such pretreatments raise the cost of the fermentation process, which contradicts the purpose of waste reuse for cost reduction^23^. The current investigation indicated the ease of utilizing the starch food wastes and used crudely for α-amylase enzyme fermentation without prior pretreatments. In the same line, *B. licheniformis* and *B. subtilis* strains were studied to degrade starch food wastes and they confirmed a high production rate with high activity quicker than pure commercial α-amylase^24^.

From the obtained results, *B. amyloliquefaciens* was selected for its α-amylase and cell mass production superiority to accomplish the current investigation. *B. amyloliquefaciens* preferred to grow and produce amylase at temperatures of 37 °C. Several studies reported the effective use of *Bacillus* species in degrading starch food wastes and α-amylase degradative enzyme activity at high temperatures^5^.

In the same line, it was confirmed that α-amylases and cell growth from *Bacillus* bacteria thrive best at neutral pH values ranging from 6.0 to 7.0, like *B.subtilis* CB-18 and *B. stearthermophilus*^25^. Furthermore, *B. subtilis*, *B. thermooleovorans*, *B. licheniformis*, and *B. amyloliquefaciens* require a pH range of 6.5–7.2 to produce α-amylases^26^.

Aeration is a vital factor in amylase production and may be controlled by altering the shaking intensity and working volume of the fermentation media^11^. This is because dissolved oxygen becomes available to the organism and, as a result, enzyme production^15^. The slow shaking speed reduced the enzyme's production substantially. Similarly, because aeration is important in the growth of aerobic bacteria^27^ observed that higher agitation increases α-amylase production^28^. Our findings were consistent with *B. licheniformis* EMS-6's optimal agitation speed for amylase activity, which was 200rpm. RSM proved these findings indicate the thermostability and alkaliphilic activity of the α-amylase enzyme^21,27^.

The calcium alginate beads were washed with distilled water and introduced to a fresh reaction starch-PBS solution mixture to examine immobilized crude α-amylase reusability. Reusing crude immobilized enzymes is critical for reducing the amount and expense of the necessary enzyme in the industrial process^29^.

Reusing of immobilized crude α-amylase at batches 13, 14, 15, 16, 17, 18, 19, and 20, the activity fell to retained only 95, 90, 83, 77, 72, 66, 61, and 55%, respectively. The activity reduction for crude α-amylase reusability may be due to natural enzyme modifications (denaturation) and enzyme adherent loss from the carrier polymer^30^. Other findings of α-amylase reusability include the use of B. circulans that produce α-amylase immobilized by entrapment in Ca-alginate beads retained 83.0%^24^ and 30%^23^ of its original activity after seven batch reuses.

Enzymes are uniquely suited as biocatalysts due to properties like complex structure, catalytic efficiency, solubility, specificity, and environmental safety. Consequently, enzymatic desizing has become a prevalent eco-friendly technique in textile processing^31,32^. It gently degrades starch-based sizing agents via enzymes like amylase, cellulase, maltase, and dextranase^33^ without damaging the fabric, unlike harsh chemical desizing^29^. In this study, alpha-amylase concentration significantly impacted the desizing of textiles, with 347.5 U/mL exhibiting maximal efficacy. Higher concentrations did not improve desizing, aligning with other reports utilizing 130–1000 U/mL amylase^27–29^.

Industrial wastewaters often have reduced dissolved oxygen from organic pollutants like starch, inhibiting treatment^34^. Alpha amylase can effectively degrade starch contaminants owing to its biochemical properties^35^. Here, a statistically significant quadratic model (F = 6.51; R^2^ = 0.83) described starch removal based on variables including pH, temperature, time, and enzyme/starch levels. All parameters substantially influenced starch hydrolysis, confirming previous observations on amylase-mediated wastewater remediation^36^. For instance, increased dissolved oxygen was reported after amylase treatment, likely due to organic matter breakdown^23^. In summary, alpha-amylase is a promising, eco-friendly biocatalyst for textile desizing, and wastewater treatment given its specificity and efficiency in starch degradation under suitable conditions. Optimization and modeling of the enzymatic process provide a framework to leverage its advantages over conventional chemical methods. As a result, crude α-amylase, immobilized by entrapped Ca-alginate beads from *B. amyloliquefaciens,* may be reused for 20 batches of repeated cycles with retained full activity.

## Conclusion

In conclusion, this study demonstrates an effective bioprocess for converting discarded bread waste into the industrially relevant enzyme α-amylase using Bacillus species. The crude enzyme displayed high activity up to 695 U/mL and stability over a wide pH and temperature range. Immobilization further enhanced reusability, with retained activity after 20 cycles. Optimization of production and application parameters enabled versatile utilization in textile desizing and wastewater treatment. Overall, upcycling abundant food waste through *Bacillus* bioconversion provides a sustainable source of α-amylase suitable for diverse harsh industrial uses. The high activity and green production process addresses both environmental and economic concerns. Further optimization to scale up this waste-to-value bioprocess presents a promising ecological solution for industries dependent on starch hydrolysis enzymes.

## Data Availability

The raw data and analyzed data used during the current study are available from the corresponding author upon reasonable request. All microbial pathogens were provided by the Agricultural Microbiology Department, Faculty of Agriculture, Ain Shams University, Cairo, Egypt, and was deposited in the following strain providers. 1. *Bacillus amyloliquefaciens* strain BT 2022 https://www.ncbi.nlm.nih.gov/nuccore/OR251122. 2. Bacillus licheniformis strain Basma87 https://www.ncbi.nlm.nih.gov/nuccore/OP547873
