# Publisher Correction: Optimizing alpha-amylase from *Bacillus amyloliquefaciens* on bread waste for effective industrial wastewater treatment and textile desizing through response surface methodology

**DOI:** 10.1038/s41598-023-47974-0

**Published:** 2023-12-04

**Authors:** Basma T. Abd-Elhalim, Rawia F. Gamal, Salwa M. El-Sayed, Samah H. Abu-Hussien

**Affiliations:** 1 Department of Agricultural Microbiology, Faculty of Agriculture, Ain Shams University, Hadayek Shoubra, P.O. Box 68, Cairo, 11241 Egypt; 2 Department of Biochemistry, Faculty of Agriculture, Ain Shams University, Hadayek Shoubra, P.O. Box 68, Cairo, 11241 Egypt

Correction to: *Scientific Reports* 10.1038/s41598-023-46384-6, published online 06 November 2023

The original version of this Article contained errors, where in some instances the ‘Bacillus amyloliquefaciens’ was incorrectly misspelt as ‘Bacillus amyloliquificiens’.

The original Article has been corrected.

